# Adolescent Male Circumcision for HIV Prevention in High Priority Countries: Opportunities for Improvement

**DOI:** 10.1093/cid/cix950

**Published:** 2018-04-03

**Authors:** Catherine Lane, Robert C Bailey, Chewe Luo, Nida Parks

**Affiliations:** 1 Office of Population and Reproductive Health, United States Agency for International Development (USAID), Washington, DC; 2 Division of Epidemiology and Biostatistics, School of Public Health, University of Illinois at Chicago; 3 HIV Section, Program Division, United Nations Children’s Fund (UNICEF), New York, New York; 4 Office of HIV/AIDS, United States Agency for International Development (USAID), Washington, DC

**Keywords:** adolescents, voluntary medical male circumcision (VMMC), male circumcision, HIV prevention, sub-Saharan Africa

## Abstract

Global experts recognize the need to transform conventional models of healthcare to create adolescent responsive health systems. As countries near 80% coverage of voluntary medical male circumcision (VMMC) for those aged 15–49 years, prioritization of younger men becomes critical to VMMC sustainability. This special supplement reporting 9 studies focusing on adolescent VMMC programming and services comes at a critical time. Eight articles report how well adolescents are reached with the World Health Organization’s minimum package for comprehensive human immunodeficiency virus (HIV) prevention in South Africa, Zimbabwe, and Tanzania, analyzing motivation, counseling, wound healing, parental involvement, female peer support, quality of in-service communication, and providers’ perceptions, and one presents models for achieving high VMMC coverage by 2021. One important finding is that adolescent boys, especially the youngest, experience gaps in their comprehension of key elements in the World Health Organization’s minimum package. Although parents, counselors, and providers are involved and supportive, they are inadequately prepared to counsel youth, partly owing to discomfort with adolescent sexuality. At the country level, deliberately prioritizing young adolescents (aged 10–14 years) is likely to achieve national coverage targets more quickly and cost-effectively than continuing to focus on older, harder-to-reach men. The studies in this supplement point to areas where VMMC programs are achieving successes and they reveal areas for improvement. Given that prioritizing adolescents will be the best means of achieving sustainable VMMC for HIV prevention for the foreseeable future, applying the lessons learned here will increase the effectiveness of VMMC programs.

The Lancet Commission on Adolescent Health’s seminal 2016 report [1] advanced the importance of strong investments in the health of the world’s adolescents and youth aged 10–24 years. A nuanced understanding of adolescence sees the adolescent brain and social interactions as molding adolescent capabilities and behaviors. As the report noted, “[i]nvestments in adolescent health and wellbeing bring benefits today, for decades to come, and for the next generation” [1].

The health needs of adolescents and young adults have been mostly overlooked in global health programs and policies, but today many countries are beginning to seriously grapple with how best to respond to adolescents’ health needs, especially in the prevention and treatment of human immunodeficiency virus (HIV) infection and for sexual and reproductive health (SRH). Global experts recommend that these investments be “inter-sectoral, multilevel, and multicomponent” and transform “traditional models of health-care … to create adolescent-responsive health systems” [1].

Most new HIV infections occur in sub-Saharan Africa, with adolescent girls and young women aged 15–24 years up to 8 times more likely to acquire HIV infection than their male peers [2]. Adolescent girls and young women often have limited capacity to negotiate safer-sex practices, such as abstinence or condom use, so reducing HIV risk in young men through voluntary medical male circumcision (VMMC) remains an important prevention option [3] and a key element of the Joint United Nations Programme on HIV/AIDS (UNAIDS) Fast-Track Strategy to end the AIDS epidemic by 2030 [4]. Better community engagement approaches and innovations for VMMC that are effective, efficient, and sustainable are needed now.

As countries near 80% VMMC coverage in 15–49-year old men [5], described as saturation, prioritization of younger men becomes critical to VMMC sustainability. Three options include (1) regular campaigns to engage communities to support VMMC service provision to adolescents, (2) introduction and scale-up of early infant male circumcision, or (3) both early infant male circumcision and adolescent VMMC, assuming less than complete coverage in infants. In any of these scenarios, adolescents are critical to achieving near-universal VMMC coverage with optimal efficiency.

Before the release of 3 randomized controlled trials demonstrating a 60% protective effect of VMMC against HIV acquisition in adult men [6–8], numerous studies of the acceptability of VMMC among adult men and women in eastern and southern African countries reported a strong preference for adolescent male circumcision, preferably before the onset of sexual activity [9–11]. Boys aged 8–15 years were cited by most respondents as the ideal priority group for VMMC, with communities believing that boys could contribute to decision making for the program, would understand the significance of VMMC, and would be able to take care of the wound themselves, and that the wound in boys would heal faster than in male adults [9–13]. 

Circumcision during adolescence was also preferred because it aligns with the age at which some communities traditionally circumcise boys [14]. Adult respondents in these acceptability studies believed that circumcision for adults or older adolescents resulted in higher risk of complications, more pain during the procedure, painful post-VMMC erections, delays in healing, and costly time away from work [9–13]. A few studies that examined adolescent perspectives found boys and girls to be familiar with and have positive attitudes toward circumcision, associating it with modernity and hygiene [11, 15]. Subsequent experience also showed that young men were more likely than older men to accept VMMC [14, 16]. Despite the availability of all this information, the World Health Organization (WHO), UNAIDS, and the US President's Emergency Plan For AIDS Relief (PEPFAR) focused initial efforts on reaching men aged 18–49 years, based on analyses that prioritizing these age groups would achieve the most rapid impact on the epidemic [17]. If adolescents aged 10–16 years had been prioritized, a high proportion of them would be circumcised. They would be 19–25 years old by now, approaching or in the years of highest HIV incidence, therefore preventing a greater number of new infections [18].

This special supplement reporting studies focusing on adolescent VMMC programming and services comes at a crucial time as countries deliberate on ways to effectively achieve sustainability of their VMMC programs. Nine timely articles analyze multiple aspects of adolescent VMMC in 3 countries: South Africa, Tanzania, and Zimbabwe. The achievement of UNAIDS’ 2021 goal of 90% VMMC coverage among boys and men aged 10–29 years [19] necessitates an understanding of how adolescents seek and perceive VMMC services and how best to support providers in delivering a high-quality service that adheres to national guidelines. Eight studies examine how well adolescents are reached with WHO’s minimum package for comprehensive HIV prevention and safe VMMC, analyzing motivation, counseling, wound healing, parental involvement, female peer support, quality of in-service communication, and providers’ perceptions. One article presents several models for achieving high VMMC coverage by 2021.

## STRENGTHS AND LIMITATIONS

To date, few VMMC studies have included adolescents as participants. The studies in this special supplement include data collected from large samples totaling up to 1526 adolescent boys and young men, as well as individuals and groups influential in adolescent VMMC decision making and/or involved in pre- and post-VMMC service delivery. The latter included parents/guardians, providers, and female peers. The investigators leading these studies represent a wide range of disciplines and perspectives, including epidemiology, the behavioral and social sciences, modeling, program implementation, and evaluation. The application of mixed methods, with standardized qualitative and quantitative data collection and analysis offering the opportunity for triangulation of results, contributes to robust findings.

As with all studies, those included in this supplement have limitations. One is that only adolescents seeking VMMC and parents/guardians of adolescents who had agreed to be or were recently circumcised were interviewed. This is understandable, because the research was intended primarily to evaluate and provide better understanding of in-service communications. However, to identify ways to create and mobilize demand among adolescents, additional research is needed to learn about those who oppose VMMC or who are still contemplating action. Future research should also focus on improving both clinical aspects of VMMC specifically for adolescents and the monitoring and evaluation of program adherence to national guidelines.

## LESSONS LEARNED

We frame our commentary using the ecological model of health behavior [20] because all the studies reported in this supplement examine VMMC from multiple levels, including perspectives from adolescent boys, parents/guardians, peers, sexual partners, health providers, the health system, and the community at large.

### Individual Level

At an individual level, adolescent boys are interested in and seek VMMC, they demonstrate comprehension gaps, and they want information on sexuality and condoms. In most programs, most VMMC clients are adolescent boys and young men aged 10–19 years, with the largest proportion of adolescent clients aged 10–14 years. For many young men, VMMC is their first independent contact with the health delivery system [21], but there has been little attention to addressing WHO’s recommendation that health programs consider the specific characteristics of adolescents [22]. Although most adolescents appeared satisfied with the services, boys in both rural and urban settings reported poorer quality of services when compared to those in periurban settings, and younger adolescents were more likely to perceive a lower quality of counseling [23]. These observations should be considered when evaluating VMMC programs, especially those prioritizing adolescents.

When researchers observed interactions, they found that counselors demonstrated respectful interest with young clients, and they used supportive body language, indicating that they were likely to be perceived as supportive by adolescents engaged in the service [24]. Nearly all adolescents (97%) reported high satisfaction with VMMC, and >72% were very likely to recommend VMMC to their peers [23].

There were gaps, however, in the information and counseling provided, particularly to younger boys. Many did not receive WHO’s full VMMC “minimum package,” although those aged 15–19 years received more package items than did boys aged 10–14 years [24]. VMMC program adherence to protocols should be closely monitored to address gaps in the response. Older adolescents were more likely to receive HIV education and testing, condom demonstrations, and condoms to take home than younger boys. Observations confirmed that condom demonstrations occurred less frequently for younger adolescents [24]. Older adolescents had greater knowledge of the importance of condom use, about having fewer sex partners, and about the importance of faithfulness [25].

WHO’s minimum package should be adapted to address the particular needs of adolescents, considering their neurocognitive development and level of maturity, as well as adolescent preferences and age-differentiated follow-up care. Parental involvement and community sensitization and engagement are also important. Kaufman and colleagues [24] suggest that all adolescents should receive comprehensive prevention messages but that younger adolescents may require less technical descriptions. Information should be easily comprehensible, relevant to the adolescent client’s experience, and delivered in ways that respect adolescent privacy and confidentiality [24, 26].

Providers paid limited attention to counseling young clients about postsurgical wound care and sexual practices [24]. Many adolescents had suboptimal knowledge of wound care, with poor recall or misinterpreted or disregarded provider instructions [26]. Many believed that pain management was poorly addressed, and they wanted greater information on condom use and the partial HIV protection provided by male circumcision. Lower knowledge levels among younger adolescents may be due to provider preferences for group-based over individualized counseling. Group counseling may be less effective for younger boys, if it is delivered alongside older boys with messaging that is not tailored specifically to the younger boys’ level of understanding. Furthermore, group counseling conducted with a mixed age group, may skew content to older boys, with younger boys remaining silent, not wishing to appear unknowledgeable.

A positive finding is that many adolescents report satisfaction with VMMC, which is not always the case for other services sought by adolescents. Adolescents frequently cite negative provider attitudes about obtaining contraception or HIV testing [27, 28].

### Family Level

Parents are important in supporting their adolescents’ VMMC decision making from the process of consenting to caring for the wound to reinforcing provider education about sexual behavior [26, 29]. The majority of adolescents reported discussing personal matters with both parents. Younger adolescents were more likely than older adolescents to report good communication with their parents, and parents were more likely to be actively engaged in VMMC with younger boys [29].

Although parents are involved, engagement seemed limited to discussing the benefits and risks of VMMC and providing consent. Parents reported an inability to communicate with their sons, especially those aged 15–19 years [29], about condom use, sexuality, and safer sex practices. Parents feared that “talking about sex would encourage their sons to engage in it.”

A WHO report [30], affirms that parents influence healthy decision making among adolescents, but many parents are unaware of how best to exert their influence, and social and/or gender norms often do not encourage open conversation about SRH between parents and sons. A study with adolescents aged 10–14 years in northern Uganda [31], found that boys had more limited social support for talking about SRH than did girls.

VMMC programs can reinforce parental influence through disseminating information, holding sessions for parents, and applying tools and resources that enhance parental skills and capability. Key approaches should strengthen parental understanding of the value of VMMC while helping them analyze their own values, norms, and beliefs. They should also correct any myths around adolescent sexuality and facilitate the development of communication and other skills that support healthy decision making.

### Facility Level

At the facility level, providers welcome adolescents but are uncomfortable with adolescent sexuality and omit information. Adolescents reported wanting information on pain, wound care, healing time, protection from HIV, the procedure itself, side effects, and condom use, with some differences by age [23]. Observations of and reports from providers revealed gaps in the information and counseling provided to adolescent clients, particularly for younger adolescent boys [21,24]. Providers hesitated to communicate sexual health information to younger boys, whom they assumed to be sexually inexperienced [21]. Many providers held back information “perceived to be irrelevant…for clients aged <15 years. These topics could be broached with adolescents *if the provider deemed it appropriate*” [21] (emphasis ours).

Providers often reflect the values and beliefs of the communities where they work; in addition to being health workers, many are also parents. They acknowledge that, despite availability of national VMMC guidelines, many independently determine counseling content based on the age and assumed sexual experience of the client. Providers underscored their need for specialized training to develop their skills on adolescent specific counseling and communication [21]. WHO has developed resources [32] for primary care providers to improve core competencies in adolescent health, and the international guidelines on sexuality education from the United Nations Educational, Scientific and Cultural Organization (UNESCO) [33] provide guidance on communicating age-appropriate SRH information.

Kaufman et al [24] propose a “checklist” to standardize improved counseling, based on the success of surgical checklists, but such a tool may not do enough to dispel provider discomfort in discussing SRH with adolescent boys and could even reinforce poor counseling skills. The authors did report that the quality of VMMC counseling improved when providers used a checklist and counseling was conducted for older men expected to be sexually active, possibly because providers were less uncomfortable when counseling this population.

### Community Level

At the community level, peer and partner support creates positive social norms, but some norms can limit VMMC. Positive social norms that reward adolescents for seeking VMMC are important to VMMC uptake, whereas negative norms that are disincentives need to be addressed. In traditionally noncircumcising communities, initial resistance to VMMC has given way a decade later to VMMC beginning to be perceived as normative. VMMC programs can be a forum to promote other positive norms and behaviors for adolescents and young men related to HIV prevention, care, and treatment, SRH, alcohol and drug use, and even gender-based violence and positive models of masculinity.

Adolescent boys, especially older ones, report HIV prevention and protection from other infections as the most common motivation to seek VMMC. Other reasons included suggestions from school officials, advice from “others,” because friends were doing it, and the wish to avoid ridicule for not conforming. About one-half of the study population believed that the majority of their friends were circumcised [34]. Peer norms, as well as those of teachers or religious leaders, clearly influence adolescent VMMC decisions. Most adolescent boys report that they expect to be circumcised, and they fear stigma from peers and girls if they are not circumcised. Younger boys are less concerned with stigma and more likely to respond to external cues, such as seeing their friends volunteering for the procedure [34].

Most parents indicated joint parent-son decision making for VMMC. In Zimbabwe, however, some parents feared VMMC, citing concerns about HIV testing and unsupportive social norms if HIV-positive status is found. Parents’ apprehension about such social censure limited their ability to communicate more supportively with their sons about VMMC [29].

As found elsewhere, women’s opinions about circumcision both positively and negatively influence men’s VMMC decisions [13]. Kaufman et al found this to be true also of adolescents. Because women benefit from VMMC through reduced risk of HIV and other infections [35], their opinions and beliefs can and should be addressed so that they encourage and support their male partners, siblings, and friends in being circumcised [36].

### Country and International Levels

At the country and international levels, predicting the impact of age-group prioritization for VMMC scale-up can guide planning and implementation of VMMC programs. Mathematical modeling provides insights into potential outcomes based on today’s programmatic decisions, including immediacy of impact, cost-effectiveness, and differences between alternative strategies for VMMC. The modeling for this supplement investigated different scenarios for increasing circumcision coverage by 2021 among men aged 10–29 years in 9 priority countries [18]. A country that has already attained high coverage among 10–14-year-olds (ie, Tanzania) has a much better chance of achieving 90% coverage among 10–29-year-olds, because historical coverage affects future coverage.

The modeling used by Njeuhmeli et al [18] does not factor in the cultural acceptability of adolescent circumcision nor the ease and lower cost of recruiting this age group. Although challenging, models should try to incorporate acceptability and ease of implementation into their estimates. That used by Njeuhmeli et al does confirm that deliberately prioritizing young adolescents (aged 10–14 years) is likely to achieve the 90% coverage targets more quickly and more cost-effectively than strategies that continue to focus recruitment on older, harder-to-reach adolescents and adults.

## CONCLUSIONS

One of the most compelling narratives across the research is the sheer number of adolescent boys and young men who are eligible for and interested in VMMC. In the 3 countries studied, adolescents and youth, ages 10–21 years, make up approximately one-third of the total population (29% in South Africa, 32% in Tanzania, and 36% in Zimbabwe [37]), with the numbers in these cohorts projected to increase in coming decades. It would be imprudent to overlook the needs of this crucial subpopulation when VMMC programs offer an opportunity to reorient services to be relevant, comprehensive, and effective for adolescents and young men.

Conventional models of healthcare delivery for adolescents [32] must become more responsive to the specific profile and ongoing needs of adolescents and their communities. The studies in this special supplement reveal areas where VMMC programs are achieving some successes, and they point to areas where improvements are needed. As the 3 countries profiled here and other VMMC priority countries strive to improve their adolescent VMMC programs, they would do well to consult these studies. It is becoming clear that approaches that better engage with and address the perceptions and concerns of adolescents, their parents, caregivers, and communities are more likely to succeed. Ensuring that programs are not driven by short-term results but are consistent with and responsive to community preferences will be crucial. 

As new innovations and approaches are introduced, ongoing monitoring and evaluation of programs to drive quality improvement consistent with national guidelines will become increasingly important. Given that prioritizing adolescents will be the best means of achieving sustainable VMMC for HIV prevention for the foreseeable future, applying the lessons learned here will be valuable for ensuring effective programs going forward. Together, these studies demonstrate both the challenges and the opportunities offered by VMMC programs to support the health and well-being of adolescent boys today and the men they will be tomorrow.

## Notes

***Supplement sponsorship.*** This article appears as part of the supplement “Adolescent Voluntary Medical Male Circumcision: Vital Intervention Yet Improvements Needed,” sponsored by Johns Hopkins University.

***Potential conflicts of interest.*** All authors: No reported conflicts of interest. All authors have submitted the ICMJE Form for Disclosure of Potential Conflicts of Interest. Conflicts that the editors consider relevant to the content of the manuscript have been disclosed.
